# Competency assessment for community health nurses: a focus group expert panel discussion

**DOI:** 10.1186/s12912-022-00898-y

**Published:** 2022-05-30

**Authors:** Ramlah Kisut, Hajah Dayang Jamilah Haji Awang Sulaiman, Hanif Abdul Rahman, Khadizah H. Abdul-Mumin

**Affiliations:** 1 Department of Nursing Services, Ministry of Health, BB3910, Bandar Seri Begawan, Brunei Darussalam; 2 Pengiran Anak Puteri Rashidah Sa’adatul Bolkiah, Institute of Health Sciences, Universiti Brunei Darussalam, BE1410, Gadong, Brunei Darussalam; 3 Research Scholar, School of Nursing, University of Michigan, Ann Arbor, USA; 4 Adjunct Senior Lecturer, School of Nursing and Midwifery, La Trobe University, VIC3086, Bundoora, Australia

**Keywords:** Competency, Assessment, Tool, Instrument, Community health nurses, Primary health care, General practice setting

## Abstract

**Background:**

General Practice setting in the Primary Health Care Services are the utmost visited by the public. It is important that the nurses’ competencies in this area be assessed to ensure provision of safe and quality services.

**Aim/objective:**

To explore perceptions and experiences of competencies assessment tool for community health nurses working at the General Practice setting in the Primary Health Care Services.

**Methods:**

An exploratory qualitative study utilizing focus group discussions were conducted on purposive sample of 12 officers with expertise in competency assessment and community health nursing from higher nursing education institutions, the Nursing Training and Development Centre, the Nursing Board and the Community Health Nursing Services in Brunei Darussalam. The existing competencies assessment tool was revised, the participants were divided into two groups of expert panel review team and two focus group discussions were held with each team. The focus group discussions encompassed components and methods of assessment; methods of grading; and overall organization and structure of the revised competency assessment tool.

**Findings:**

Four themes emerged: 1) International equivalent core competencies components; 2) Multi-methods approach to assessment; 3) Definitive guidelines as framework for assessment; and 4) Understanding and acceptability of the competency assessment tool.

**Conclusions/implications to practice:**

The expert panel reviews provide practical input that were inculcated in the preliminary developed competencies assessment tool. Identification of eligible assessors were recommended based on standardized criteria, and socialization and training held to set direction and guidance for implementing the utilization of the competencies assessment tool. Further studies are deemed important to critically evaluate and validate the preliminary competencies assessment tool for development of a more robust assessment instrument.

**Supplementary Information:**

The online version contains supplementary material available at 10.1186/s12912-022-00898-y.

## Introduction

Healthcare systems across the globe are equally and primarily aiming at providing quality and safe health care to the public. It is important for the front-facing healthcare providers, for example such as nurses [1], midwives, and social workers to have the appropriate competencies at least at a minimum standard to enhance healthcare provision which is of quality and safe care.

The worldwide perspectives of professional nursing competencies are focused on providing safe and quality service. It is the responsibility of each professional nurse to be competent in delivering the skills sets required to improve and sustain the quality of patient care, hence increasing patient satisfaction [2]. Substantial evidence also pinpointed the importance for health care organizations to give attention to professional competencies for nurses to maintain safety and quality service [3]. Medical errors, negligence, or malpractice built from incompetence can risk patients’ lives [4]. For all these reasons, there is a need for valid and reliable instruments to assess the competencies of nurses in the practice setting. Many attempts to define competence and competencies have been published in the literature but there are still confusion and lack of clarity surrounding the concept [5]. Competencies can be described as a combination of observable and measurable knowledge, skills, abilities, and personal attributes that constitute an employee’s performance. Whatever the agreeable definition is, the ultimate goal is that the employee can demonstrate the required attributes to deliver safe and quality care [6].

## Background of the study

### The importance of competencies standard

In the healthcare system, core competency standards are the standards and requirements usually set by the relevant healthcare profession regulatory body to inform standards of practice of the specific profession intended for the provision of safe and quality care to the public as consumers of the healthcare system. Such healthcare professions extend beyond nursing and include for example midwifery, social workers, physicians, optometrists [7], and other medical professions [8]. Core competency standards are important for these healthcare professions for monitoring of safe practice and provision of quality patients’ care.

In the nursing profession, nursing regulatory body is responsible for nurses’ competencies in providing safe and quality care to patients. Having competent nurses in the healthcare services increase trust and confidence of the public [9–11]. Being competent in providing patients’ care also uphold the high reputation of nursing profession [12]. Poor and unsafe patient care may occur if nursing practices do not met expectations laid in the core competency Standards [13, 14].

### Context of the study

Nursing profession in Brunei Darussalam (henceforth: Brunei) is the largest that contributed to at least 70% of the total healthcare workforce [15]. There are different rank of nurses working at various clinical sites in the three level of healthcare system of Brunei – Primary, Secondary and Tertiary. The first level of health care system provision in Brunei is the Primary Health Care that comprised of 14 primary health centers and 16 maternal and child clinics. There are about 300 CHNs working in 14 PHC across Brunei. To work as CHNs, they are required to have at least an education background of general nursing. Whereas, those who work in maternal and child clinics are required to have midwifery or community health nursing qualifications. Maternal and child health (MCH) nursing, or midwifery, or Community Health Nursing qualifications are additional advantage. Some CHNs work at the General Practice (GP) setting in the PHC providing care in the outpatients’ departments (OPDs), Chronic Diseases Clinics (CDCs), and nurse-led clinics. Other CHNs work at the MCH setting providing maternal and child health care, and Women’s Clinics. Both GP and MCH settings are integrated in the PHC. This paper specifically focuses on the CHNs in the GP setting.

### Core competency standards in Brunei

Similar to other countries, in Brunei Darussalam (henceforth: Brunei), there also exists core competency standards for Registered Nurses developed by the Brunei Nursing Board (BNB) [16], the national regulatory body that governed nursing practice. However, the core competency standards are more generic but not specific to nurses working in a specific setting. One of the aims documented in the core competency standards is:

*“to assess clinical performance competency and measure the professions’ fitness to continue practice in Brunei Darussalam”.*

[16].

Aligned with the core competency standards for Registered Nurses, a generic competency assessment tool (GCAT) was also developed by the Brunei Nursing Training and Development Centre (Pusat Latihan Perkembangan Kejururawatan [PLPK]). However, the GCAT is generic and only purposely designed for assessing newly employed nurse, for a one time use when they were first employed. CAT for continuous assessment of core competency standards for Registered Nurses are not available yet. Nurses in Brunei are assessed based on evaluation of the Key Performance Indicators (KPIs) assigned by the Department of Nursing Services via the Head of Nursing in each department across the primary, secondary and tertiary level of healthcare. This KPIs are integrated feature in the yearly performance appraisal tool (PAT) designed by the Department of Public Services which are generic for all civil servants’ regardless of their professions. The PAT may only be suitable for assessing and evaluating generic skills, which pose challenges to the nursing profession as it is not specific to nurses’ skills and competencies.

Another CAT was developed in 2016 by a Primary Health Centre (PHC) [17], which is specific and initially used biennially for assessing core competency standards of the community health nurses (CHNs) working at General Practice in the PHC. It is identified that this CHNs CAT was developed in response to the PHC accreditation assessment by the Joint Commission International. Since then, there is no further evidence of regular and continuous use of this CHNs CAT.

### Limitations of the existing CHNs CAT

The development of the CHNs CAT were not based on a systematic process of instrument development and validation, and the core competencies assessed may risk the bias views of the former developer(s). The tool has never been reviewed and evaluated, hence, reliability and validity of the CATs have yet to be established. Assessment of core competency standards should not be a one off, but a regular and continuous process so that standards are consistently maintained throughout nursing practices. This concurs with the international health care accrediting agencies such as Joint Commission International [6] which highlighted the requirement for periodic performance evaluation to assure ongoing competencies of nurses.

In terms of the feature and contents of the CHNs CAT, analysis uncovered that the instrument only predominantly contains a set of basic nursing procedural skills to be assessed by checking the ‘DONE’ or ‘NOT DONE’ column (See Table 1 for examples of skills assessed). Other higher level of core competencies such as that recorded in the BNB [16] are not found. These include legal and ethical framework of practice, professional practice, leadership and management, continuous professional and personal development, and education and research. There is no scale that differentiate achievement of a CHN to another. The method of assessing was only through observation methods instead of diverse methods of evidence, to name a few the CHNs CAT do not use such as reflective accounts, critical incidence analysis, peer review, evidence-based practice and acknowledgement from patients and colleagues at work.

**Table 1:** Examples of what were assessed in the existing CHNs CAT

1. Wound dressing	
2. Triaging outpatient cases	
3. Infection prevention and control	
4. Immunization	
5. Collecting specimen for pap test	
6. Computer information system	
7. Electrocardiogram	

### Needs and significance of the CHNs CAT development

In view of the limitations of the CHNs CAT that assessed only generic, basic and practical nursing skills, being the Head of the Community Health Nursing Services in Brunei, the primary author takes the lead for evaluation, development, implementation and validation of the CHNs CAT as a pilot or trial. The similar process may further be adapted in the development of CAT in other nursing clinical areas. This paper presents the first two of the four-part tasks which are the instrument evaluation and development.

It should be noted that the role of nurses in GP setting has expanded over the years [6]. With the evolving and expansion scopes of nurses working in the GP setting of the PHC, CHNs CAT need to be developed in alignment with the expanded scope of practices (SoP) and consistent with the actual regulated practices. Reviewed of literature evident that nurses practices either below or beyond their SoP that cause either loss of or inadequate skills that eventually will compromise nursing practices in the General Practice setting of the PHC [18]. Hence, this study is significant in various ways: 1) consideration of development of a credible and reliable CHNs CAT that is appropriate and able to accurately assess competencies of CHNs working at the GP setting in the PHC; 2) in a longer term, this study can inform development of similar standardized CAT for assessing core competency standards of nurses in many different clinical settings in Brunei.

### Aim/objective

This study aimed to explore expert panel perceptions and experiences of CHNs CAT for CHNs working at the General Practice setting in the PHC Services in Brunei Darussalam. The objectives were:

1. To evaluate the competencies required for CHNs working at the GP setting in the PHC Services
2. To determine how would the competencies be assessed
3. To develop preliminary CHNs CAT

## Research design and methods

### Design

This study was qualitative and exploratory underpinned by instrument evaluation and development process, utilizing focus group discussions to collect in-depth data on expert panel’s perceptions and experiences of competencies assessment tool for CHNs working at the General Practice setting in the Primary Health Care Services.

### Ethical considerations

This study is conducted in line with the principles underpinning the Declaration of Helsinki [19]. Ethical clearance was provided by the joint committee of the Pengiran Anak Puteri Rashidah, Institute of Health Sciences Research Ethics Committee (IHSREC), Universiti Brunei Darussalam and Medical and Health Research and Ethics Committee (MHREC), Ministry of Health (ERN: UBD/PAPRSBIHSREC/2O19/18). Written permission to evaluate and re-developed the existing CAT was granted by the Director of Health Services and Director of Nursing Services. Participants were assured that their participations were voluntary and they could withdraw from the study without penalty at any time throughout the study prior to completion of data analysis. Written informed consent was obtained from all participants once their enquiries were answered and they were fully satisfied with the information about the study. Confidentiality was ensured where the study was done in private room free from distractions and access of others. Participants’ anonymities were also ensured where participants were coded using a personal identification number (PIN code). They were requested to refer to this code and were not allowed to call real names during the FGDs. The research and any publications will not report participants’ details that can easily identified their affiliations.

### Preparation prior to data collection

#### Defining CHNs CAT

A working definition for CHNs CAT was formulated to ensure all the study participants have the same idea on the CHNs CAT, hence, guide them in the evaluation and development. CHNs CAT is defined as an instrument or a tool for assessing and evaluating CHNs competencies that encompassed their knowledge and skills which should be performed at a minimum standard.

#### Identification of literature for instrument evaluation and development

The research team conduct literature search on evidence-based core competencies standard where four international regulatory documents were initially gathered to facilitate expert panel review. Table 2 illustrated the key documents.

**Table 2:** International regulatory documents

1)General Practice Nurse Competencies from the United Kingdom (Royal College of General Practice Foundation and Royal College of Nursing [20])	
2)Primary Health Care Competency Framework originated from Canada (Capital Health Nova Scotia [21])	
3)National Practice Standards for Nurses in General Practice from Australia (Australian Nursing and Midwifery Federation [22])	
4)World Health Organization, Competencies for nurses working in Primary Health Care [6].	

### Data collection

#### Expert panel review

Expert panel or working group was formed. Participation and selection as expert panel were by invitation where letters were sent to the Dean and Directors of the higher nursing education institutions, Head of Brunei Nursing Board, Head of Nursing Administration of the Community Health Nursing Services, and Head of the Nursing Training and Development Centre for nomination of at least an expert in developing CAT for CHNs at the General Practice setting in the Primary Health Care Services. The mixture of different participants ensured a diverse range of stakeholders within the specialization of either community health nursing or competency assessment. The participants were selected using purposive sampling guided by coherent inclusion criteria (Table 3). Participants whom do not met these criteria were excluded.

**Table 3:** Inclusion criteria for the study

For participants to be eligible as expert panel in the study, they must have:	
1)knowledge and/or experiences of either the domains, skills and job descriptions of community health nurses in General Practice settings of the PHC; or competency assessment tool development;	
2)been working clinically for at least 5 years in their field.	

Through FGDs the expert panel critically review, analyse, compare, evaluate and synthesize the existing CHNs CAT with the current empirical evidences from the authoritative regulatory documents. The components and/or domains on competencies standard of CHNs in the existing CHNs CAT was compared with those in the international regulatory documents. The expert panel further suggested appropriate recommendations for development of the preliminary revised CHNs CAT. The expert panel also added another three international regulatory documents that further facilitate evaluation and development of the preliminary revised CHNs CAT:

1. International Council of Nurses (ICN), Nursing Care Continuum Framework and Competencies [9]
2. The Nursing Council of Hong Kong, Core competencies for Registered Nurses (General) [23]
3. Jordanian Nursing Council, National Standards and Core Competencies for Registered Nurse [11]

It is expected that expert panel review tasks will be conducted only by one expert panel group throughout the instrument evaluation and development to ensure consistencies in the development of the preliminary revised CHNs CAT. Six participants initially volunteered in the expert panel review. However, due to other administrative commitments, it was not possible to get the same expert panel to wholly complete the tasks, hence invitation was extended, and another six participants further volunteered which formed the second group of expert panels. The change in the planning was observed as opportunistic as it considers the diverse views [24] from the two expert panel groups. They comprised of nursing academics from the higher nursing education institutions, nursing managers of Community Health Nursing Services from the PHC Services, nursing authorities from the Nursing Board and competencies evaluators and trainers from the Nursing Training and Development Centre.

Four FGDs, two for each team were held with the expert panel review team. The first FGD was to collect analysis, evaluation, perceptions and experiences related to the revised competencies assessment tool from the first expert panel review team, and a second one was conducted to finalize their collective agreement. The third FGD was held with the second expert panel review team to determine if there were any further divergent feedbacks which may be overlooked by the first team, and the last one was to finalize all feedbacks from the second team for development of a preliminary revised CHNs CAT. The FGD encompassed components and methods of assessment; methods of grading; and overall organization and structure of the revised competency assessment tool.

The revised CAT was emailed to the expert panel a week before the FGDs to allow them preparation prior to the FGDs. The FGDs were also guided with a pre-designed open-ended question so that the FGDs would not side track. All FGDs were audio recorded with consent from the participants to ascertain accurate and consistent account of the FGDs for transcriptions.

Table 4 presented summary of the critique of the existing CAT by the two expert panel group. The domains and performance indicators were identified and refined which resulted to construction of a revised version of the preliminary CHNs CAT.

**Table 4:** Critique of the existing CHNs CAT

Criteria	Current CAT	Suggested recommendation from the literature
Competency tool development process	Methodology not reported in the document	Comprehensive literature reviews are critically important for gathering evidence-based practice [24] on CHNs CAT. Methodology for instrument development includes involvement of panel experts through focus Group Discussion (FGD), Delphi approach o Face-to-face interview which should be done in a systematic process (see Table 2). The instrument must be pretested [24].
Methods/approaches used for assessment of competencies	Limited to direct observation of skill demonstrated by assessors	Adopt variety of assessment approaches. E.g. return demonstration, case presentations, case studies, certification recognized by the nursing profession, continuing education programmes related to the nurses individual practice, documentation review, examinations for skill assessment and/or clinical reasoning, nursing Research, skill assessment inventories (via self, peers, supervisors, and clients), observation of daily work, portfolio development and review, presenting at local, state and national meetings, publishing in a scholarly journal, quality improvement indicators, self-directed learning activities, Self-assessment tools, and simulations [13, 25].
Performance indicators	Tick boxes limited to two performance indicators (either DONE or NOT DONE)	Competency involves more than just technical and procedural skills [13]. Use findings from the literature and international regulatory documents.
Level of competence	Not acknowledge	Adopt strong theoretical background [24] such as Benner’s framework of a competent nurse – from novice to expert to acknowledge level of competence [26].
Competencies assessed	Limited to procedural skill Other aspects of competencies such as thought process (critical thinking skill) and knowledge, communication and values are not assessed	Based on criteria such as high risk, low volume, problem prone procedures or situations, unusual incidents and regulatory requirements. Should also focus on new, changing, high-risk and problematic area of practice [25].
Frequency of competency	Not stated in the document	On-going and assessed at regular interval as identified by the organization [8, 27].
Who performed the competency assessment	Not mentioned in the document	Should be registered nurses skilled in the field of competence assessment and methodologies to ensure valid and reliable competency assessment processes [8, 28].

### Data analysis

All FGDs were transcribed verbatim. The transcriptions were then checked for accuracy against the audio recordings. Two members of the research team (first and last authors) systematically analyzed the transcripts to identify both deductive themes based on the focus group guide and inductive themes that emerged during coding. Transcripts were read and re-read to identify potential themes. Emerging themes were then coded by hand independently by each team members, then coding was compared with discrepancies resolved through discussion to enhance reliability of the findings.

## Results

### Participants’ characteristics

Participants were six nurse managers from Community Health Nursing Services, two academics from the higher nursing education institutions, two authorities from the Nursing Board and two senior nurses with capacities as competencies evaluators and trainers from the Nursing Training and Development Centre. Nine of the participants were female (75%) and the rest were male (25%). All of these individuals have expertise in both competency assessment and Community Health Nursing services. Their details and main expert field is presented in Table 5. They have more than 10 years of clinical work experiences in community health nursing setting of the PHC and are well versed with the core competencies of a CHN. For confidentiality purpose, gender, exact age and qualifications, and workplace are not reported.

**Table 5:** Expert Panel Characteristics

Participants’ Personal Identification Number	Main Expert field	Age range	Qualification
P01	Competency Assessment	26–35 years	Degree
P02	Community Health Nursing	36–45 years	Higher Degree
P04	Competency Assessment	46–55 years	Higher Degree
P05	Community Health Nursing	26–35 years	Degree
P06	Competency Assessment	36–45 years	Diploma
P07	Community Health Nursing	46–55 years	Degree
P08	Competency Assessment	26–35 years	Degree
P09	Community Health Nursing	36–45 years	Higher Degree
P10	Competency Assessment	46–55 years	Higher Degree
P11	Community Health Nursing	36–45 years	Degree
P12	Competency Assessment	46–55 years	Degree

### Findings from the expert panel review

Table 6 showed some examples of the results derived through the process of deductive and inductive coding of the FGDs transcripts that eventually lead to the finalization of themes. Quotes that exemplified the inductive coding are presented in the final themes. Four final themes emerged from the expert panel review process of refining and finalizing the preliminary CHNs CAT: 1) International equivalent core competencies components; 2) multi-methods approach to assessment; 3) Definitive guidelines framework for assessment; and 4) Understanding and acceptability of the competencies assessment tool.

**Table 6:** Examples of deductive and inductive coding

Deductive coding	Inductive coding	Finalized Themes
Components of core competencies to be assessed	Benchmark with international regulatory documents	Theme 1: International equivalent core competencies components
	At par with international countries	
	Ensure Brunei CHNs can function globally	
	Different CATS for different level of nurses	
	Target for Staff Nurse only	
	Contains generic competencies and also specific competencies for CHNs	
	Must not be procedural only – should contain more than basic and procedural skills	
	Cover and demonstrate higher level of thinking	
	Leadership and management must be assessed because CHNs working in GP setting are like nurse specialist or practitioners	
	Identify domains or components, put what are to be assessed under the components or domains	Theme 3: Definitive guidelines as framework for assessment
	Describe performance indicators, what needs to be achieved, how it needs to be achieved, how achievements are measure	
Methods of assessment	Must be varieties	Theme 2: Multi-method approach to assessment
	Do not use observation only	
	CAT should be assessor/assessors friendly	
	Use reflective diaries	
	Evidence of research/evidence-based practice	
	Objective structured clinical examination/ assessment	
	Case studies/case presentations	
Methods of grading	Scale must be not too wide and not too rigid	Theme 3: Definitive guidelines as framework for assessment
	Define the scale, give descriptions of the scale	
	From novice to expert	
	Four or seven scale	
	Audit	
	Viva defense/verbalization/discussion	
Organization and structure of the CAT	Readable	Theme 4 – Understanding and acceptability of the competencies assessment tool
	Terms used must be understood by both assessors and assesses – provide glossary of terms	
	Socialization of the CAT through workshops and seminars	
	Acceptable to be implement (not westernized) - culturally acceptable	
	Feasible in Brunei healthcare system	

### Theme 1: international equivalent core competencies components

This theme described the expert panel affirmation for the core competencies components to be equivalent of the international standards. All of the expert panel pointed out that the core competencies components should be benchmarked that equivalent to the international standards. The expert panel justified that this is to ensure that the CHNs will achieve the minimal core competencies standards which should be arranges into key components. The World Health Organization (WHO) is the most commonly referred international organization as the governing body for the CHNs core competencies.

“ … the competencies tool should follow competency framework from WHO. It should be at the international standards so that performances of our community health nurses should be at par with other countries … The core competencies should be divided into five clusters and under each cluster there should be list of competencies to be achieved” (P04, Expert Panel Group 1, FGD 1)

Majority of the expert panel also pointed out that benchmarking of the core competencies standards should be comparable with the requirement of the International Council for Nurses competencies framework [9]. They also pinpointed that the core component of competencies also needs to reflect role of PHC nurses in General Practice or also called Out-Patient Department (OPD) setting, and should be consistent with the core competencies standards set by the Nursing Board for Brunei.

“ … the components of the competencies should mirror the ICN competencies framework but also must matched with NBB (Nursing Board for Brunei) requirements. Comparing these both together, the core competencies components should be put into domain. For examples, ethical responsibilities, leadership or continuous professional development, and so on. Then it will be easy to arrange the competencies either skills or knowledge under each domain” (P02, Expert Panel Group 1, FGD 1)

Arranging core components into key areas or domains was agreeable by the expert panel to provide clarity of the knowledge or skills set under the domains. Five core competency standards (Legal and ethical framework for practice; professional practice, leadership and management; continuous professional and personal development, and education and research) established by Nursing Board for Brunei [16] were commonly suggested by majority of the expert panel.

“ … The ICN core competency standards are extended version. But core competency standards from NBB are succinct. We should use the five main components and arranged list of competencies under these five main components accordingly” (P01, Expert Panel Group 1, FGD 1)

“ … It would be more appropriate if we adopt competency standards from local context … so it would be meaningful as we also teach our student using these core competency standards.” (P05, Expert Panel Group 1, FGD 1)

### Theme 2: multi-method approach to assessment

This theme explained the expert panel assertion that the assessments grading system should not be rigid to observations only but diverse encompassing other methods such as audit, certificate of training and chart review. It was notified by some of expert panel that method of assessment in the revised CAT need more clarification in terms of appropriate methods of assessment that will accordingly assessed and measure specific competencies performance.

“How do we assessed a specific skill required by the specific core competencies components? We cannot depend on 100% observations only. Assessing through discussion with others may be subjective too. The main point is the appropriateness of the competencies assessment, it should assess what it should measure. For examples achievement of skills require direct observations, demonstration of knowledge require evidence of assignments, and research may need evidence such as published manuscripts or evidences of changes in practices … ” (P10, Expert Panel Group 2, FGD 3

The expert panel believed that evidences of competencies should be included or submitted at the end of assessment period to ensure validity of the assessment conducted. The expert panel provided examples of evidences such as certificate of attendance or participation, audit result, chart review or other relevant documentation supporting the achievement of the core competencies. Quality improvement activity was also suggested by half or the expert panel to diversify methods of assessment. Other expert panel members also recommended that Objective Structured Clinical Examination (OSCE) as on the best way to assess competency albeit time consuming.

“ … I think OSCE is a good way to assess competency but we may not able to afford it … It need time and lots of resources in preparation for the session” (P12, Expert Panel Group 2, FGD 2)

Many participants favour the use of different methods of assessment over single method;

“ … apart from the stated methods, can we add quality improvement activity as one of the assessment methods? . . . it can save much of our time to assess some of the components by just providing evidences of participation or contributions such as certificates, letter of acknowledgment or participation, and so on. This should be submitted to support the competencies assessment. This is to make sure that the community health nurses truthfully achieved the performances which were assessed.” (P6, Expert Panel Group 1, FGD 2)

### Theme 3: definitive guidelines as framework for assessment

This theme represented the expert panel emphasis on the importance of a distinctive grading system that can differentiate the performance of newly employed nurses from the experienced nurses. All of the expert panel were on favor of a scoring or grading system for the competencies assessment.

“The grading system or scoring system for competencies assessment is very good. It gives high marks to high performer nurses and low marks to low performer nurses. It is good because it differentiates how a nurse is more competent than the others, and remedies can be planned to improve competencies.” (P09, Expert Panel Group 2, FGD 3)

However, about three quarter of the expert panel recommended that in view of the multiple methods of assessment, explanations should accompany the grading system as a framework that guide the grading or scoring system. They commented that development of such framework will be useful because the General Practice or OPD is usually a very busy setting, hence, if the CAT is unclear, the purpose of doing competency assessment will be defeated by time constraint, work overload, inadequate staffing and lack of knowledge on how to use the CAT among assessors and the nurses to be assessed.

“ … the use of different methods of assessments on the same competencies is very good. The direct observations may be complemented by collections of reflective diaries, which further can be strengthen by providing certificate of attendance that sharpen the skills being assessed. However, how do we know the assessor is choosing the right method of assessments for a particular performance in the core competencies component, while other assessor may also use different method for that same performance?” (P12, Expert Panel Group2, FGD 3)

A few of the expert panel argued that due to the scale nature (1 to 5) of the grading system, there may be issues in segregating how the score be awarded to an experienced nurse from the new nurses.

“ … I am not a 100% supportive of the grading system … an assessor may not have adequate knowledge on how to rate the performance … again the different methods of assessment that can be employed … also because the scale is only 1, 2, 3, 4 and 5. How would you rate based on this scale to an experienced nurse and how would you differently rate a new graduate nurse?”(P11, Expert Panel Group 2, FGD 3)

The concern about the possibilities of inconsistencies among assessors were also highlighted by a quarter of the expert panel as assessment can be subjective reliant on the individual assessor.

“ … different nurse managers may have different way of interpreting their competency assessment findings so at the end of the day we may have discrepancy of the score given” (P08, Expert Panel Group 2, FGD 3)

“ … some nurse managers may be very lenient, but some may strictly adhere to their high level of expectation … … this again all depend on their individual interpretation of the performance standards.” (P05, Expert Panel Group 1, FGD1)

### Theme 4 – understanding and acceptability of the competencies assessment tool

This theme illustrated the expert panel concern about the users’ understanding of the CAT comprising the nurse assessing and the nurse to be assessed in order to ensure that expected performances are similarly perceived by both parties. Majority of the expert panel pointed out that competency standards should be appropriately assessed by an experienced or senior nurse. They further highlighted that assessment needs to be done regularly as an ongoing activity in order to monitor and maintained the standards of practice.

“ … The competencies assessment should not be a one-off activity … looking at the number of components, we must set interval period for the assessment to be conducted … are we going to make it annually or every 3 years … ” (P07, Expert Panel Group 2, FGD 4)

More than half of the expert panel felt that the CAT acknowledged their understanding of the CAT addressing that it will be useful to assist nurse managers in determining whether or not a community health nurse is competent in a particular standard. Having said that, the expert panel also proposed several recommendations to be put in place before the implementation of the CAT. It was perceived that the CAT can be utilize properly with adequate information and guidance along with adequate training, particularly on what are the expectations on the competency standards nurses have to achieve and how to utilize the CAT.

“ … I can see that this CAT can be useful to ensure nurses are competent though it may be very tough to conduct the assessment if nurse managers are not fully informed about the assessment. The CAT must be clear in every aspect so that the nurse who assessed and the nurses to be assessed equally understand expectations laid on by the CAT. A briefing and training on how to use the CAT would be a good start before using the CAT in practice … ” (P03, Expert Panel Group 1, FGD 2)

Three quarters of the expert panel expressed their acceptability of the CAT and stated that the revised CAT would be more applicable and useful than the existing generic annual performance appraisal for civil servant. They raised the issue of time constraint and increase workload, if the CAT would be additional to the performance appraisal.

“ … I can foresee the difficulty face by nurse managers if the CAT is used in addition to annual performance appraisal establish for civil servant. It will be extra work for nurse managers and some of us may not have enough time to do them both at one time” (P08, Expert Panel Group 2, FGD 3)

## Discussion

This study was conducted to explore expert panel perceptions and experiences on the existing CHNs CAT including its components, methods of assessment, grading system and its overall structure and organization. This study evident that it is fundamental to identify a comprehensive core competencies domain and the list of core competencies for CHNs in the general practice setting of the PHC Services. This was pinpointed by the expert panel in their critique of the existing CHNs CAT as shown in Table 6 and theme 1 of the study findings. As emphasized by the WHO [6], the role of nurses in GP setting has expanded over the years [6]. Identification of a set of clear competencies domains ensure that CAT assessed the scope of practices (SoP) within the country they practice, hence, consistent with the actual regulated practices [18]. The findings are consistent with qualitative research exploring the relevance of existing Australian Competency Standards for Registered Nurses that is capable of assessing the specific community health nursing practices. CHNs must be better equipped with knowledge, skill and ability to deal with these complexities in order to provide safe and the best care possible.

It is important to highlight that competencies are acquired and developed steadily and progressively overtime, which should not be a ‘one off’ or ‘once-only’ activity. Assessment should be continuous, on-going and perform at regular interval [8]. This is particularly true in a way that it allows time for assessors to adequately observe, monitor and evaluate the performance rather than jump into conclusion at 1 hour observation for instance. The issue of ‘once-only’ assessment and a ‘tick-box’ approach in competency assessment should be given attention as there is empirical evidence to show that these strategies may not be able to adequately assess competence [8, 24, 27, 29].

Review of the CATs internationally also suggested that the core competencies standards should be expanded to include higher level of knowledge and skills such as research, leadership and management [30]. Our study demonstrated that the seven-core competency generic nurses standards framework advocated by the ICN [9] appropriately aligned with the five-core competency generic nurses standards domains established by the Nursing Board for Brunei [16]. Comparison of the two generic competencies standards with other international countries showed that the contents are all similar and relevant [11, 23, 31, 32]. Likewise, the specific core competencies standards for CHNs in the preliminary revised CHNs CAT concurs with Brunei Nursing Board [16] and the rest of the international regulatory documents.

The alignment of the preliminary revised CHNs CAT with the local and international document coincide with the expert panel view that implied CAT should be developed based on benchmarking with international regulatory bodies and other countries. It is viewed that benchmarking ensure that core competencies of the CHNs in Brunei are at least at the international standards. This is expressed by the expert panel in theme 1. This may possibly due to the fact that community health services worldwide are facing similar current global challenges. These include growing number of chronic diseases, ageing population and shortage of healthcare professionals [33]. Aside from this, another possible reason is due to the global mobility and migration of nurses that requires them to acquire competencies which are globally adaptable [34].

The findings presented in this study delineated the process of instrument evaluation and development undertaken by the expert panel that include critical analyses and evaluations of the existing CHNs CAT, which were further revised, refined and finalized by the expert panel through the process of FGDs. This process was conducted systematically and is considered rigorous in terms of face and contents validity [35]. Future studies should consider quantitative design encompassing pilot testing of the CAT and performing the psychometric properties for determining reliability and validity of the preliminary CHNs CAT. The systematic process in instrument development which was first advocated by Benson and Clark [36] and evident in many of the developed instruments include the implementation and psychometric properties testing for determining instruments’ reliability and validity. It is anticipated that the CHNs CAT for the general practice setting of the PHC Services would set the basis for assessment to determine the competence level of CHNs from entry into practice and throughout their professional nursing careers instead of a one-off activity.

Concern over the inconsistencies of perceptions of the CAT from the FGDs with the expert panel are also consistent with findings from the literature [29]. Traditional approach of competency assessment is distorted with ambiguity and inconsistency. Such concern may be justified because competencies assessments are subjective to individuals whom may either be lenient or have high expectations. In addition, interpretations of assessors are subject to clarity of the core competencies to be assessed. The expected core competencies must be communicated to the CHNs and their assessors so that they have similar understanding of the competencies, hence the competencies performance and assessment are conducted as expected [37, 38].

In term of acceptability to use in practice settings, time constraint, work overload, inadequate staffing and lack of knowledge on how to rate competence are the identified obstacles that may lead to hesitancy to use the preliminary CAT. These findings are similar to a study conducted by Figueroa, et al. [39] in determining the compliance of nurses to national core competency standard. The use of multi-methods and multi-assessors approaches in conducting assessment may solve this issue. Holanda et al. [40] indicates that using these approaches may reduce inconsistencies among assessors as well as reduce time taken to do the assessment. Although evidences on the most effective method is limited, there is general agreement in the literature that competency assessment should use more than one assessment methods that include such as self -assessment, direct observation, Objective Structured Clinical Examination (OSCE), and simulation [24, 29]. The multi-methods competencies assessment and multi-assessors of core competencies acknowledged uniqueness of individual nurses through diverse approaches. Other widely used method is patient-centered competency model which addressed patient as an assessor to add greater reliability and validity to the assessment process [29].

This study pinpointed that usability and acceptability of using the preliminary CHNs CAT additional to the existing generic civil servant’s performance appraisal may be viewed as task duplications by assessors, in particular, nurse managers which is highlighted in theme 4. The use of multiple competency assessment tools to meet mandatory evaluation of performance and regulatory requirements may put extra burden for the assessing nurses and the nurses being assessed alike. This may at the end highly likely resulted to the ‘tick-box’ approach which defeat the purpose to adequately assess nurses ongoing ability to competently undertake their daily nursing duties [40]. In an equilibrium, being specifically competent of nursing practices denotes safety and quality measures in nursing care whilst annual appraisal will only look at the general performance of aptitude and attitude nursing staff.

### The preliminary CHNs CAT

The revised version of the preliminary CHNs CAT was refined and finalized as Draft 1 Preliminary CHNs CAT (see supplementary materials) after three major revisions following the expert panel review from the FGD. Upon suggestion from the expert panel review, the preliminary CHNs CAT now contents glossary of the terms used in the CAT, a concise introduction of the document, components of the competencies, method of assessment, grading system and a step-by-step guide on how to use the document. The expert panel equally agreed for the importance of culturally specific and context specific CHNs CAT, hence the inclusion of the additional three documents.

The expert panel also aligned the requisites identified in the international regulatory bodies with the core competencies standards of the Brunei Nursing Board [16] in the preliminary CHNs CAT. The whole process of developing the CHNs CAT is not completed yet. Instrument development should include pilot testing the CAT and analysis of the psychometric properties of the CHNs CAT for its reliability and validity. The next tasks for the primary author is to continue on implementation, validation, and reevaluation and redevelopment of the CHNs CAT accordingly. It is planned that these will be done in the near future.

## Conclusion

This study had provided two distinct inputs. First, the valuable insight in the refinement of a preliminary developed CAT and second, the identification of issues that may affect the acceptability and the implementation process of the revised CAT. The expert panel had given substantial contributions in the revision process of the existing CAT leading to preliminary development of a new CAT. The study highlighted the significant of using a multi-method and multi-assessors’ approach in the assessment. These include direct observations of the nurse’s practice, an interview to ascertain nursing care in different scenarios and evidences provided by the nurse (including self-assessments, exemplars or examples of practice, documentation, and reports from other nurses and other health professionals). Adequate information and training of using the preliminary new CAT, and providing clear explanations of terms used in the preliminary new CAT are some recommended solutions concerning the utilization and acceptability of the CAT. It is also suggested that competencies assessments should be periodic, regular assessment instead of a ‘once-only’ assessment. It is found imperative to paid attention to multiple competency assessment tools which may put extra burden to the nurses.

### Strengths and limitations of the study

This study reviewed the existing CHNs CAT for nurses working at the General Practice setting in PHC through the review of expert panel groups that have background in community health nursing. The FGDs with the expert panel evident that challenges remain in establishing components or domains of competencies, list of competencies and assessments approaches in terms of methods and assessors. Further FGDs may deem required to establish a more comprehensive list of assessment items. Further quantitative analysis is also required to assess the psychometric properties of the preliminary new CAT to evaluate the tool critically, hence development of a more robust assessment instrument.

### Implication to practice, policy and research

The World Health Organization (2010) emphasize that it is imperatively important to ensure nurses are competence to perform their jobs. The American Joint Commission on Accreditation of Healthcare Organization (AJCAHO) (2010) claimed that in order to provide quality patient care, the individuals delivering patient care services must be competent enough to do so. AJCAHO standards also require leaders to ensure the competence of staff members to be continually assessed, maintained, demonstrated and improved [8]. Therefore, taking safety and quality of care into consideration, it is vital to utilize reliable and validated tool to assess competency. Policy makers may enforce the preliminary revised CHNs CAT for implementation.

There is no single best method that can be used to assess competence. The combination approach is recommended to ensure adequate assessment of competence. Methods of assessment identified in the literature include return demonstration, skill assessment inventories (via self, peers, supervisors, and clients), portfolio development and review and observation of daily work [25, 41]. Thus, this reflects that using ‘one size fits all’ approach poses a significant limitation to competency-based assessment in the clinical setting which demonstrated that one single method will not be able to adequately measure competence. Future research should include this area to consistently improve the preliminary revised CHNs CAT.

It is worth mentioning that the revised CAT is intended to assess and evaluate competency standard at staff nurse level. Knowing the importance of assessing and evaluating competencies of other level of nurses, it is imperative to develop similar tool with different core competencies standards in the future studies.

## Supplementary Information

**Additional file 1.**

## Data Availability

The datasets generated during and analyzed during the current study are not publicly available due to institutional data sharing policy but are available from the corresponding author on reasonable request.

## Abbreviations

AJCAHO: American Joint Commission on Accreditation of Healthcare Organization
CATs: Competency Assessment Tools
CHNs: Community Health Nurses
FGDs: Focus Group Discussions
IHSREC: Institute of Health Sciences Research Ethics Committee
ICN: International Council of Nurses
MHREC: Medical and Health Research and Ethics
OPD: Outpatient Department
OSCE: Objective Structured Clinical Examination
PHC: Primary Health Care
PLPK: The Brunei Nursing Training and Development Centre
SoP: Scope of Practice
WHO: World Health Organization
