# Supplementary material for: Competency assessment for community health nurses: a focus group expert panel discussion
Source: BMC Nurs. 2022 May 30;21:134. doi: 10.1186/s12912-022-00898-y (PMC9150315; doi:10.1186/s12912-022-00898-y)
Supplement: Supplementary file 1 — Additional file 1. [file 12912_2022_898_MOESM1_ESM.docx]

**COMMUNITY HEALTH NURSING SERVICES**

**COMPETENCY ASSESSMENT TOOL FOR S­­­­­TAFF NURSES**

**IN GENERAL PRACTICE OF THE PRIMARY HEALTH CARE**

**COMPETENCY FRAMEWORK DEVELOPMENT TEAM**

Lead by:

Ramlah Kisut, Special Grade Nursing Officer, Head of Community Health Nursing Services

Expert panel reviewers (Contributors): **(Name not mentioned for confidential reason)**

1. Name, Nursing Officer, area of work
2. xx, Nursing Officer, xx
3. xx, Nursing Officer, xx
4. xx, Staff Nurse, xx
5. xx, Nursing Officer, xx
6. xx, Senior Staff Nurse, xx
7. xx, Staff Nurse, xx
8. xx, Staff Nurse, xx
9. xx, Nursing Officer, xx
10. xx, Nursing Officer, xx
11. xx, Academic, xx
12. xx, AAcademic, xx

**INTRODUCTION**

This document is intended to be used as a guideline for evaluating the key competencies of nurses in Primary Health care (PHC) in general practice outpatient setting which specifically focuses on the Staff Nurse level. This competency assessment framework addresses the specific competencies and the wider range of skill, knowledge and ability a nurse need in this setting in order to be fully proficient and competent. It is also acknowledge that assistant nurses (ANs) and other registered nurses at different level are also essential part of primary health care teams and foresee their scopes of practice will be included in future development of similar resources. The framework is complimentary to, and supported by, the following documents:

1. Code of Ethics for Registered Nurses and Midwives in Brunei Darussalam (2013). Nursing Board for Brunei, Ministry of Health
2. Code of Professional Conduct for Registered Nurses and Midwives in Brunei Darussalam (2013). Nursing Board for Brunei, Ministry of Health
3. Core Competency Standards for registered Nurses and Midwives in Brunei Darussalam (2013). Nursing Board for Brunei, Ministry of Health

This competency assessment tool (CAT) has been developed with reference to a number of key frameworks from international nursing institutions/ organizations including:

1. International Council of Nurses (ICN), Nursing Care Continuum Framework and Competencies (2008)
2. World Health Organization, Competencies for nurses working in Primary Health Care (2015).
3. Royal College of General Practitioners (RCGP) General Practice Foundation / Royal College of Nursing (RCN), General Practice Nurse Competencies (2015)
4. National Practice Standards for Nurses in General Practice from Australia (Australian Nursing and Midwifery Federation, 2014)
5. Primary Health Care Competency Framework originated from Canada (Capital Health Nova Scotia, 2012)
6. The Nursing Council of Hong Kong, Core competencies for Registered Nurses (General) (2012)
7. Jordanian Nursing Council, National Standards and Core Competencies For Registered Nurse (2016)

**How can the framework be used?**

1. It provides a detailed picture of the main role of a nurse working at general practice setting in Primary Health Care. The competencies are specifically related to roles and responsibilities of nurses at Staff Nurse level.
2. It is designed as an initial assessment as well as ongoing monitoring and evaluation tool to help individuals recognize their current level of competence and identify specific areas for further development. This is to ensure that individuals recognize gaps in their knowledge and work within the scope of professional practice (Nursing Board for Brunei, Ministry of Health 2013).
3. During the preceptorship / training period it can be used as a tool to review and demonstrate progress, recognize the acquisition of specific skills and knowledge and provide evidence of assessment of safe clinical practice. A record of how the evidence of competence was demonstrated and achieved should be included.
4. It can form the foundation of a portfolio of continuing professional development to assist all nurses regularly review their level of competence and ensure they continue work within their scope of their professional practice.
5. It is recommended as a tool to support the process of annual appraisal and provide evidence of meeting the required standard stipulated by Nursing Board for Brunei
6. The document can also inform and support the design and delivery of education and training and nursing workforce planning

**COMPONENTS OF COMPETENCIES**

The framework is designed to identify the competencies required by Staff Nurse level to fulfil a variety of roles within general practice setting in Primary Health Care. The competencies comprise of five domains which are in line with Nursing Board for Brunei Core Competencies Standards for Nurses and Midwives in Brunei Darussalam (2013). These domains include:

- Core Competency Standard 1: ***Legal and Ethical Framework of Practice***
- Core Competency Standard 2: ***Professional Practice***
- Core Competency Standard 3: ***Leadership and Management***
- Core Competency Standard 4: ***Continuous Professional and Personal Development***
- Core Competency Standard 5: ***Education and Research***

Each domain is elaborated further into specific descriptions statement of performance indicators that need to be assessed using appropriate method of assessment.

**METHOD OF ASSESSMENT**

Reviews conclude that there is no generally accepted ‘gold standard’ for the assessment of competence. Therefore, a multi-method approach to assessment is recommended to optimize reliability and validity which include:

- Direct observation of the nurse’s performance
- Return demonstration of specific procedure/ clinical tasks
- Question and answer sessions to assess underpinning knowledge
- Reflective discussion between the nurse and the assessor regarding professional progress
- Case study presentation to demonstrate understanding and knowledge on specific competencies
- Written test to assess required knowledge for certain aspects of nursing care
- Documented nursing care through patient chart review
- Nursing audit to review patient’s record to identify, examine or verify the performance of certain specified aspects of nursing care with the set standard.
- Certification

Method of assessment (refer to MOA table) used by the assessor is put under the column MOA on the Competence Assessment Tool and the score is entered under the column ‘Rating Score’. The grading system of scale from 1 – 4 (refer to Grading system table) is used to guide the assessor in the performance rating. In the event that the nurse does not meet the minimum required standard (i.e. score of 1 in an identified performance indicator/s, improvement action plan should be drawn up and agreed between the nurse and the assessor.

| **Method of Assessment (MOA)** | |
| --- | --- |
| **A** | Audit |
| **C1** | Certification |
| **C2** | Case presentations / Case studies |
| **D** | Documentation / Chart review |
| **E** | Objective structured clinical examination/ assessment (OSCE / OSCA) |
| **O** | Observation/ Return Demonstration |
| **P** | Presentation at local/ departmental / ministerial level |
| **T** | Written test |
| **V** | Verbalization / Discussion |

The improvement action plan must be documented and should detail the following:

1. Agreement on the part of the Nurse and the Assessor as to the exact area/s where a problem/s are identified
2. Specific details of how the problem area/s will be addressed in the clinical area
3. An agreed period of time for re-assessment

A full review and further development of the improvement contract and action plan will also be required if the nurse is deemed incompetent after the re-assessment of the identified areas.

**GRADING SYSTEM**

| **PERFORMANCE** | **RATING SCORE** | **DESCRIPTION** |
| --- | --- | --- |
| **Excellent (A)** | **4** | Performs **beyond** which would be expected **at his/her level of experience:**  - Display a high level of understanding of the procedure / performance - Demonstrate advanced knowledge on the topic / performance  discussed  - Outstanding performance and exceed the requirement needed to  perform safely and effectively |
| **Very Good (B)** | **3** | Performs **better than** what would be expected **at his/her level of experience:**  - Display a high level of understanding of the procedure / performance - Meet the requirement needed to perform safely and effectively  - Able to describe / explain the procedure with full details |
| **Good (C)** | **2** | Performs **well** as expected **at his/her level of experience:**  - Display a good understanding of the procedure / performance  discussed with some minor weaknesses  - Able to describe / explain the procedure well  - Meet the required standard but may need slight improvement of  minor weaknesses to perform effectively |
| **Unsatisfactory (D)** | **1** | Performs **below** what is expected **at his/her level of experience:**   - Does not meet the minimum required standard - Demonstrate limited / lack of knowledge of the procedure - Require attention to improve the performance to perform safely |

| **NO** | Performance Indicators | **MOA** | **Rating Score** |
| --- | --- | --- | --- |
| 1 | ***DOMAIN 1: LEGAL AND ETHICAL FRAMEWORK FOR PRACTICE*** | | |
| 1.1 | Compliance to the national legislation, policies and guidelines that have legal implications on practice including BruHims policy and guidelines; and Official Secrets Act (OSA) |  |  |
| 1.2 | Maintain privacy and confidentiality of patient information all the times |  |  |
| 1.3 | Competently perform accurate documentation procedures |  |  |
| 1.4 | Practice in accordance with the Code of Ethics, Code of Professional Conduct and Standards of Practice for Registered Nurses & Midwives in Brunei Darussalam, legislations of Brunei Darussalam and the Malay Islamic Monarchy philosophy |  |  |
| 1.5 | Demonstrate awareness of the legal and ethical issues pertinent to practice setting: accountability, duty of care, breaches of privacy and confidentiality of patient’s information and informed consent |  |  |
| 2 | ***DOMAIN 2: PROFESSIONAL PRACTICE*** | | |
| **2.1** | **Fundamental and Principles of Primary Health Care (PHC)** | | |
| 2.1.1 | Describe the principles of PHC i.e. providing health promotion and disease prevention, early detection and intervention, and maintenance of health to improve health outcomes of individual, family, group, community and/or population |  |  |
| 2.1.2 | Identify current national health priorities and practices in a manner consistent with the fundamental and principles of primary health care. |  |  |
| **2.2** | **Assessment of Patient** |  |  |
| 2.2.1 | Utilize PHC triage guidelines for management of patient |  |  |
| 2.2.2 | Obtain accurate subjective assessment such as patient complaint, pain/ discomfort, relevant family history, previous surgical history, social history and presence of co-morbidities |  |  |
| 2.2.3 | Competently conduct objective assessment such as vital signs and other required assessment |  |  |
| 2.2.4 | Recognize abnormal physical assessment findings, prioritize and responds appropriately |  |  |
| 2.2.5 | Use effective communication to handover assessment findings: verbal (ISBAR handover) and written form (clinical notes) |  |  |

**PERFORMANCE INDICATORS**

| 2.3 | **12 Lead ECG and interpretation.** | | |
| --- | --- | --- | --- |
| 2.3.1 | Able to initiate and undertake a 12 Lead ECG |  |  |
| 2.3.2 | Able to recognize anomalies of ECG reading and respond promptly |  |  |
| 2.4 | **Wound Care** | | |
| 2.4.1 | Perform initial assessment of patient presenting with wound |  |  |
| 2.4.2 | Perform comprehensive wound assessment including wound classification in order to formulate care plan |  |  |
| 2.4.3 | Perform wound irrigation and apply a range of dressing according to assessed need |  |  |
| 2.4.4 | Assess wound closure and perform suture removal as indicated |  |  |
| 2.4.5 | Provide adequate health education to manage wound at home and identification of red flags |  |  |
| 2.4.6 | Schedule for follow-up appointment accordingly |  |  |
| 2.4.7 | Evaluate wound healing progress   - Identify factors affecting wound healing - Recognize signs and symptoms of infection and delay wound healing and other indication that need referral - Able to plan, implement and evaluate wound care |  |  |
| **2.5** | **Immunization** | | |
| 2.5.1 | Adhere to vaccine administration Standard Operating Procedure (SOP) including preparation, administration and post vaccination procedure. |  |  |
| 2.5.2 | Accurately document in BruHIMS and patient CDC booklet/ immunisation book as per guideline. |  |  |
| 2.5.3 | Awareness of national vaccination program |  |  |
| 2.5.4 | Awareness of Adverse Event Following Immunisation (AEFI) reporting form and adhere to procedure for reporting of AEFI as per guideline |  |  |
| 2.5.5 | Adhere to cold chain maintenance according to SOP including daily record of fridge temperature, safe vaccine storage and procedure for transporting of vaccines |  |  |
| 2.5.6 | Act accordingly in the event of break in cold chain (adherence to break in cold chain SOP and reporting procedure) |  |  |
| 2.5.7 | Demonstrate adequate knowledge in the management of anaphylaxis |  |  |

| **2.6** | **Code Blue Management** | | |
| --- | --- | --- | --- |
| 2.6.1 | Able to identify individual roles in Code Blue management by applying local guidelines in the management of emergency cases |  |  |
| 2.6.2 | Adhere to SOP of monitoring and updating emergency trolley and perform regular monitoring as per guideline |  |  |
| 2.6.3 | Demonstrate adequate knowledge in the management of Code Blue |  |  |
| **2.7** | **Health Promotion** |  |  |
| 2.7.1 | Assess the health needs of the patients and provide them with information and education that enable them to promote health, assume self-care at different stages of their lives; and to cope with Non-Communicable Diseases (NCDs) and injuries which include:   - Physical Activity - Healthy eating - Smoking cessation and available support services to support smoking cessation - Compliance to medication - Compliance to schedule appointment / follow-up - Self-care and disease management plan |  |  |
| 2.7.2 | Perform skills and competence in health promotion through lifelong learning and adopt various health promotion strategies that help people to build capacity in controlling their own health and making healthy life choice |  |  |
| 2.7.3 | Facilitate and empower individuals, families and communities to increase control over the determinants of health via capacity building strategies such as common risk factors of Cardiovascular disease (CVD) including modifiable and non-modifiable risk factors |  |  |
| 2.7.4 | Tackling multiple health determinants such as CVD risk assessment by utilizing appropriate / available assessment tool and refer accordingly as per guideline |  |  |
| 2.7.5 | Contribute and participate in the implementation of health promotion strategies in partnership with other interested parties, as nursing acknowledges inter-sectoral contribution to health promotion such as  help patient acknowledge Hypertension as a risk factor for atherosclerotic vascular disease and potential end-organ damage:   - Signs/symptoms of hypotension and hypertension - Self-monitoring of BP - Benefit of physical activity and weight loss (for overweight/ obese patients) |  |  |
| 2.7.6 | Contribute and participate in the implementation of health promotion strategies in partnership with other interested parties, as nursing acknowledges inter-sectoral contribution to health promotion such as  improve health outcomes of patient with Chronic Obstructive Pulmonary (COPD) and Asthma including:   - Factors that can trigger / worsen the condition - Prevention strategies - Asthma control and management including advice on inhaler technique, measure peak flow readings and make appropriate referral where necessary - Recognition and management of acute exacerbations of asthma |  |  |
| 2.7.7 | Contribute and participate in the implementation of health promotion strategies in partnership with other interested parties, as nursing acknowledges inter-sectoral contribution to health promotion such as improve health outcomes of patient with Diabetes including:   - Self-monitoring Blood Glucose (SMBG) - Diabetes foot care & referral to Diabetes Nurse Educator (DNE) for annual foot screening - Fasting during month of Ramadhan - Acute emergencies: hypoglycaemia and Diabetic Ketoacidosis (DKA) |  |  |
| 2.7.8 | Contribute and participate in the implementation of health promotion strategies in partnership with other interested parties, as nursing acknowledges inter-sectoral contribution to health promotion such as  cancer prevention and early identification of cancer through referral for:   - Cervical cancer screening - Breast mammogram - Colorectal cancer screening |  |  |
| 2.7.9 | Evaluate the outcome of health promotion activities and pursue continuous improvement |  |  |
| 2.7.10 | Participate in and contribute to the development of evidence-based practice in health promotion |  |  |
| 3 | ***DOMAIN 3: LEADERSHIP AND MANAGEMENT*** | | |
| 3.1 | Work collaboratively and/or in partnership with other disciplines and sectors in promoting the health of the community |  |  |
| 3.2 | Understand the roles of individuals working within PHC and communicate effectively with other multidisciplinary teams to deliver people-oriented health promotion, disease prevention and continuity of care. |  |  |
| 3.3 | Establish, build, and nurture relationships with other health professionals to promote maximum participation and self-determination of individual, family and community. |  |  |
| 3.4 | Undertake mentoring of students and less experienced nurses, provide professional mentorship for and appropriately delegate clinical tasks to Assistant Nurses (ANs). |  |  |
| 4 | ***DOMAIN 4: CONTINUOUS PROFESSIONAL AND PERSONAL DEVELOPMENT*** | | |
| 4.1 | Engages in Continuing Nursing & Midwifery Education (CNME) program to update knowledge and improve skills relevant to practice setting. |  |  |
| 4.2 | Keep abreast with innovative advancements/ evidence-based and best practices.  . |  |  |
| 4.3 | Contributes to and participates in Continuous Professional Development (CPD) programs, preceptorship, coaching and mentoring to assist and develop colleagues. |  |  |

| 5 | ***DOMAIN 5: EDUCATION AND RESEARCH*** | | |  |  |
| --- | --- | --- | --- | --- | --- |
| 5.1 | Participate in various activities as a member of the research team. | | |  |  |
| 5.2 | Conduct or contribute to clinical research to continuously improve standards of care. | | |  |  |
| 5.3 | Educate self with knowledge in clinical research. | | |  |  |
| **Remarks:**  **Improvement action plan:** | | | | | |
| **Name of staff** | |  | **Signature of staff** | |  |
| **Name of assessor** | |  | **Signature of assessor** | |  |
| **Date of Assessment:** | | | **Date of expiration:** | | |

**COMPETENCY RE-ASSESSMENT (If applicable)**

| **No.** | **Performance indicators** | | | **MOA** | **Rating**  **Score** | |
| --- | --- | --- | --- | --- | --- | --- |
|  |  | | |  |  | |
|  |  | | |  |  | |
|  |  | | |  |  | |
|  |  | | |  |  | |
|  |  | | |  |  | |
|  |  | | |  |  | |
|  |  | | |  |  | |
| **Remarks:**  **Improvement action plan:** | | | | | | |
| **Name of staff** | |  | **Signature of staff** | | |  |
| **Name of assessor** | |  | **Signature of assessor** | | |  |
| **Date of re-assessment:** | | | | | | |

**GLOSSARY**

| **Glossary of terms** | **Definition** |
| --- | --- |
| Assessment | A systematic procedure for collecting qualitative and quantitative data to  describe progress and ascertain deviations from expected outcomes and  achievements |
| Assistant Nurse | Assistant nurses (ANs) in Brunei are prepared at a Certificate level (minimum educational requirement) and work under the direction and supervision of the registered nurse. |
| Competence | The combination of skills, knowledge, attitudes, values and abilities that underpin effective performance as a nurse. |
| Competency | A defined area of skilled performance which encompass the on-going development of an integrated set of knowledge, skills, attitudes, and judgments enabling one to effectively perform the activities required in a given task or function to the standards expected in knowing how to be in various and complex environments and situations. |
| General practice setting | Refer to outpatient services. General practice is the first point of contact with health care system for individuals across the lifespan. It acts as an entry point and referral portal to other providers within the health care system. |
| Nursing Board for Brunei (NBB) | This is the regulatory authority responsible for the registration of nurses in Brunei Darussalam. Its primary function is to protect the health and safety of the public by ensuring nurses are competent and fit to practice. |
| Performance indicators | Detailed actions of competent performance which assist the assessor when using their professional judgment in assessing nursing practice. |
| Scope of practice | Roles, functions, responsibilities, activities and decision-making capacity that individuals within that profession are educated, competent and authorised to perform. |
| Staff Nurse | Used interchangeably with the term registered nurse, a person licensed to practice nursing under the relevant state or country regulation and who has completed nursing training in a Nursing Institution accredited by the Nursing Board for Brunei. Staff Nurses in Brunei are prepared at a Diploma level (minimum educational requirement). |
